# In Silico Transcriptomic Analysis for Identification of Potential Diagnostic and Prognostic Biomarkers and Therapeutic Targets in Cervical Cancer using a Hybrid Genetic Algorithm–Support Vector Machine Approach

**DOI:** 10.34172/aim.34814

**Published:** 2025-12-01

**Authors:** Leila Nezamabadi Farahani, Anoshirvan Kazemnejad, Mahlagha Afrasiabi, Leili Tapak

**Affiliations:** ^1^Department of Biostatistics, Faculty of Medical Sciences, Tarbiat Modares University, Tehran, Iran; ^2^Department of Computer, Hamedan University of Technology, Hamedan, Iran; ^3^Modeling of Noncommunicable Diseases Research Center, Institute of Health Sciences and Technologies, Hamadan University of Medical Sciences, Hamadan, Iran; ^4^Department of Biostatistics, School of Public Health, Hamadan University of Medical Sciences, Hamadan, Iran

**Keywords:** Biomarkers, Cervix neoplasm, Genetic algorithm, Gene expression, Support vector machine

## Abstract

**Background:**

Cervical cancer is the leading malignancy among women worldwide, posing clinical and public health challenges. This *in silico* study aims to identify potential diagnostic biomarkers, therapeutic targets, and prognostic markers associated with cervical cancer through integrative bioinformatics approaches.

**Methods:**

A hybrid machine learning approach, combining genetic algorithm (GA) and support vector machine (SVM), was applied to high-dimensional gene expression data from publicly available transcriptomic datasets, including the Gene Expression Omnibus (GEO) and The Cancer Genome Atlas (TCGA). A total of 72 Geo samples (Affymetrix, Illumina) served as the primary dataset after normalization.

**Results:**

The GA-SVM model achieved about 99% accuracy and AUC with 10-fold cross validation, clearly separating cervical cancer from normal tissues. Eight genes (CXCL9, CTGF, ZNF704, ZEB2, SASH1, PTN, KPNA2, SLC5A1) were identified as diagnostic biomarkers. Protein-protein interaction (PPI) and functional enrichment analyses revealed 42 therapeutic targets (e.g. CDK1, BRCA1, CCNB1, and AURKB) linked to regulating cell cycle, DNA repair, and mitotic processes. Survival analysis identified six genes (CXCL1, DNMT1, MMP1, MYBL2, PCNA, and RRM2) as key prognostic markers. Additionally, transcription factor analysis identified E2F1 and TP63 as major regulators of the prognostic genes, elucidating the molecular mechanisms underlying cervical cancer progression.

**Conclusion:**

The identified gene signatures may serve as candidates for hypothesis generation and provide a computational framework to prioritize biomarkers and therapeutic targets in cervical cancer. However, these findings are based on *in silico* analyses and require experimental and clinical validation before translation into practice.

## Introduction

 Cervical cancer ranks as the second most prevalent cancer among women globally.^1^ The onset of this cancer is closely linked to persistent infection with the human papillomavirus (HPV).^2^ Approximately 120 HPV types have been identified to date, which are classified based on their oncogenic potential into high-risk and low-risk categories. The high-risk types, such as HPV16 and HPV18, are more likely to cause cancer, while the low-risk types, including HPV6, HPV11, and HPV40, are less likely to lead to malignant transformation.^2,3^ Globally, HPV16 is responsible for approximately 57% of cervical cancer cases, with HPV18 contributing to around 16%. However, the prevalence of specific HPV types in cervical cancer varies across different regions.^4^

 Interestingly, not all HPV infections lead to cervical cancer. Research has shown that nearly 90% of HPV infections clear up on their own within two years.^5^ However, the reasons behind the resolution of HPV infections in some cases and the persistence in others remain unclear. Individual susceptibility factors may contribute to the varying outcomes of HPV infections.^6^

 Currently, surgical procedures like conization or loop electrosurgical excision are the primary treatments for patients with pre-cancerous lesions or early-stage cervical cancer.^7,8^ These methods aim to remove abnormal tissue and prevent further progression of the disease. However, there is still a critical need for improved diagnostic approaches that can facilitate early detection and provide a better understanding of the molecular basis of the disease.

 Recent advancements in bioinformatics tools have facilitated large-scale analysis of transcriptomic data, enabling systematic biomarker discovery in cervical and other cancers.^9-12^ Most previous studies relied on conventional approaches such as statistical tests or single-classifier machine learning models for gene selection and diagnosis.^13,14^ However, these traditional methods, including t-test, fold-change analysis, and univariate regression, may overlook complex, non-linear relationships in gene expression data, limiting their diagnostic potential.^15,16^ To address these limitations, more sophisticated machine learning models including support vector machines (SVMs), random forests, and other classifiers have been applied to high-dimensional datasets.^17-19^ Among these, hybrid metaheuristic-ML approaches such as genetic algorithms (GA) combined with SVM have demonstrated improved effectiveness for feature selection and classification tasks, enabling more comprehensive exploration of feature space and identification of informative biomarkers.^11,20^

 Nevertheless, the use of such hybrid methods in cervical cancer studies is still limited, and many published works do not integrate these approaches with downstream functional analyses, such as protein-protein interaction (PPI) network construction and enrichment assessment.

 The primary objective of this study is to identify novel key genes that can be used as biomarkers for cervical cancer diagnosis by utilizing a hybrid GA-SVM approach. By employing these advanced machine learning techniques, the study aims to (1) enhance early detection accuracy and offer new insight into the genetic pathways involved in cervical cancer, (2) evaluate the diagnostic accuracy of GA-SVM in distinguishing tumor from normal samples (3) identify potential therapeutic targets through PPI network and enrichment analyses, and (4) determine prognostic markers using survival analysis by Gene Expression Profiling Interactive Analysis (GEPIA) platform. Ultimately, this approach could lead to more effective screening and personalized treatment strategies for individuals at risk of developing cervical cancer.

## Materials and Methods

###  Study Design, Data Acquisition, and Preprocessing

 We performed a comprehensive search of the Gene Expression Omnibus (GEO) database using the keyword “Cervical cancer” to identify pertinent datasets. The selection criteria were: (1) inclusion of primary cervical cancer and normal samples; (2) each group comprising over 20 samples; and (3) datasets encompassing more than 10,000 genes. Consequently, three microarray datasets GSE29570, GSE7410, and GSE52903 were incorporated into this study.

 Among them, GSE52903, containing 55 cervical tumor samples and 17 exocervical control samples, was used as the main dataset, while GSE29570 (45 tumor, 17 normal) and GSE7410 (40 tumor, 5 normal) served as validation datasets. These datasets can be accessed at the NCBI GEO via the following link: https://www.ncbi.nlm.nih.gov/geo/query/acc.cgi.

 To further validate our findings, we utilized the GEPIA web server, which provides access to RNA sequencing expression data from The Cancer Genome Atlas (TCGA) and the Genotype-Tissue Expression (GTEx) projects.^21^ Specifically, we analyzed the cervical squamous cell carcinoma and endocervical adenocarcinoma (CESC) dataset from TCGA, comprising 306 tumor and 13 normal samples.^22^

 For each platform, we retrieved the raw data corresponding to the three selected datasets. Subsequently, all datasets were normalized as necessary through quantile normalization using the bestNormalize package in R. We assessed the raw data for logarithmic fold change values and, when required, applied a log2 transformation. Probe identifiers were mapped to gene symbols based on the respective annotation platforms. For genes represented by multiple probes, we calculated the average expression value to obtain a single gene expression measure. Probes lacking data were excluded from the analysis.

###  Identification of Differentially Expressed Genes (DEGs)

 Differential gene expression analysis was conducted on the GSE52903 dataset to identify DEGs between primary tumors and liver metastasis samples using the limma package (3) in R. A |log₂ fold change| ≥ 1 and a false discovery rate (FDR) < 0.05 were established as the threshold for significant gene expression differences.

###  Gene Ontology (GO) and KEGG Pathway Enrichment Analysis

 To explore the biological functions of the DEGs, we performed KEGG (Kyoto Encyclopedia of Genes and Genomes, http://www.kegg.jp) and GO enrichment analyses on the selected DEGs using the ClusterProfiler (4) and GOplot (5) packages in R. Statistical significance was determined based on a Benjamini-Hochberg adjusted *P* value threshold of < 0.05.

###  Exploring Diagnostic Biomarkers Using Hybrid Machine Learning Algorithms

 In this study, we employed a hybrid machine learning pipeline for biomarker discovery, integrating feature selection and classification. A GA was used to search for optimal subsets of genes; during the GA optimization process, an SVM classifier served as the fitness function, evaluating the classification performance (e.g. accuracy) of each candidate subset in distinguishing tumor from normal samples. This hybrid GA-SVM approach ensured the identification of gene sets most relevant for accurate classification. After the GA-SVM feature selection process, the final selected features were used to train and evaluate classifiers using both SVM and an artificial neural network (ANN), allowing direct comparison of their diagnostic performance. Details of each algorithm and their implementation are described in the following sections.

###  Support Vector Machine 

 SVM is known for its ability to handle both linear and non-linear classification tasks by transforming the feature space using a kernel function.^23^

 The SVM decision function defines the boundary that separates the classes and is expressed as:

*f(x) = W.ϕ (X) + b*

 where *W* is the weight vector that determines the orientation of the decision boundary, *ϕ(X)* represents the feature mapping function that transforms the input features *X* into a higher-dimensional space to make non-linear relationships separable, and *b *is the bias term that shifts the decision boundary.

 In training the SVM model, the goal is to find the optimal decision boundary by minimizing an objective function that balances maximizing the margin width (distance between support vectors) and minimizing the classification error. The optimization problem is formulated as:


$$\min\frac{1}{2}{\left\|w\right\|}^{2}+C\sum_{i=1}^{n}\left(\xi_{i}+\xi_{i}^{*}\right)$$

subject to:


$$y_{i}.\left(W.\;\phi \left(X\right)+b\right)\geq 1-\xi_{i},\;\xi \geq 0$$

where y_i_ represents the class label ( + 1 or −1) of the i-th sample, *ξ_i_* are slack variables that allow the model to tolerate some misclassifications to improve generalization in non-linearly separable cases, and *C* is the regularization parameter that controls the trade-off between maximizing the margin and minimizing the classification error.^23^

 By solving this optimization problem, SVM identifies the hyperplane that best separates the data classes, even in complex scenarios with overlapping data points.^23^

###  Genetic Algorithm for Feature Selection

 GA is a heuristic optimization technique inspired by the process of natural selection.^24^ In this context, we used GA to explore the feature space and identify an optimal subset of features that contribute the most to the classification task. Each solution (chromosome) represents a binary vector, where 1 indicates the selection of a feature, and 0 indicates its exclusion.

 The fitness function used to evaluate each chromosome was based on the performance of the SVM classifier, measured using metrics such as accuracy and mean squared error. The GA operations (selection, crossover, and mutation) were applied to evolve the population toward better solutions over successive generations. The aim was to minimize the classification error while selecting the most informative subset of features.^24,25^

###  Artificial Neural Networks 

 The neural network was constructed with one hidden layer and trained using backpropagation, a widely used algorithm for optimizing neural network weights.^26^

 The architecture of the neural network was optimized using grid search to determine the best number of neurons, learning rate, and activation functions. To prevent overfitting, techniques such as dropout regularization and early stopping were employed.^27^

###  Cross-Validation and Evaluation Metrics

 Both the SVM and neural network models were evaluated using 10-fold cross-validation to ensure robustness and generalizability of the results. The dataset was divided into 10 subsets, and the model was trained on 9 subsets while being tested on the remaining one. This process was repeated 10 times, and the average performance was recorded.

 The models’ performance was assessed using standard classification metrics such as accuracy, precision, recall, F1 score, and area under the ROC curve (AUC), providing a comprehensive comparison between the SVM-GA and ANNs models.

 By utilizing GA for feature selection and comparing the performance with ANNs, our approach efficiently reduced the dimensionality of the feature space and improved classification accuracy. The SVM-GA model demonstrated competitive performance, making it a viable alternative to neural networks for the classification task.

###  PPI Network Analysis for Identifying Therapeutic Targets

 Genes selected more than 3,000 times across the hybrid models were utilized to construct the PPI network. PPI data were obtained from the Search Tool for the Retrieval of Interacting Genes (STRING) database (https://string-db.org). DEGs with a high confidence score (combined score > 0.7) derived from active sources, including experimental data, databases, co-expression analyses, and others, were incorporated into the network.

 The network was visualized using a spring-embedded layout algorithm, designed to optimize node placement by minimizing edge crossings and overlaps between nodes (genes).^28^ Key network metrics, such as node degree, betweenness centrality, and clustering coefficient, were calculated using the built-in tools in Cytoscape and employed as the criteria for gene selection.

###  Survival Analysis of Hub Genes for Identifying Prognostic Biomarkers

 In our study, survival analysis was performed to identify genes with potential prognostic significance. This analysis focused on the genes identified in the previous step through the PPI network. To further investigate the association between hub gene expression and cervical cancer prognosis, we utilized the GEPIA platform for survival analysis, employing the log-rank test for statistical evaluation. A *P* value of < 0.05 was considered statistically significant. The hub genes identified through this process were regarded as key prognostic markers for CESC.

###  Validation of Hub Genes’ Expression Levels

 Expression data from GEPIA was used to assess the expression levels of the prognostic hub genes identified in the previous step, comparing cervical cancer samples to normal tissues. The results were visualized through boxplots. Additionally, to investigate the differential protein expression of these prognostic hub genes, immunohistochemistry images from the Human Protein Atlas (HPA) database (http://www.proteinatlas.org) were analyzed to differentiate between normal cervical tissues and cervical tumor samples.

###  Construction of Transcription Factor-DEG Network for Prognostic Genes

 To identify the transcription factors (TFs) regulating the key genes with prognostic value, we utilized the NetworkAnalyst online tool. NetworkAnalyst is a web-based platform for comprehensive gene expression profiling and meta-analysis through network-based visual analytics.^29^ Genes with prognostic value were submitted to NetworkAnalyst to gather information on TF-gene interactions. The resulting datasets were then exported to the Cytoscape software (version 3.10.3) for further analysis. This network provides insight into the regulatory mechanisms governing the expression of prognostic genes, offering a deeper understanding of their potential role in disease progression.

###  Software and Reproducibility

 All computational analyses were performed using MATLAB (version R2021b) and R (version 4.4.2). Specific R packages included limma, clusterProfiler, GOplot, and bestNormalize. The Cytoscape software (version 3.10.3 and v.3.8.2) and the NetworkAnalyst online platform were also utilized for network-based analyses and visualizing PPI and TF–DEGs Interaction network. All custom scripts and codes are available from the authors upon reasonable request.

## Results

###  Screening Cervical Cancer-Associated DEGs in the Datasets

**Figure 1:**
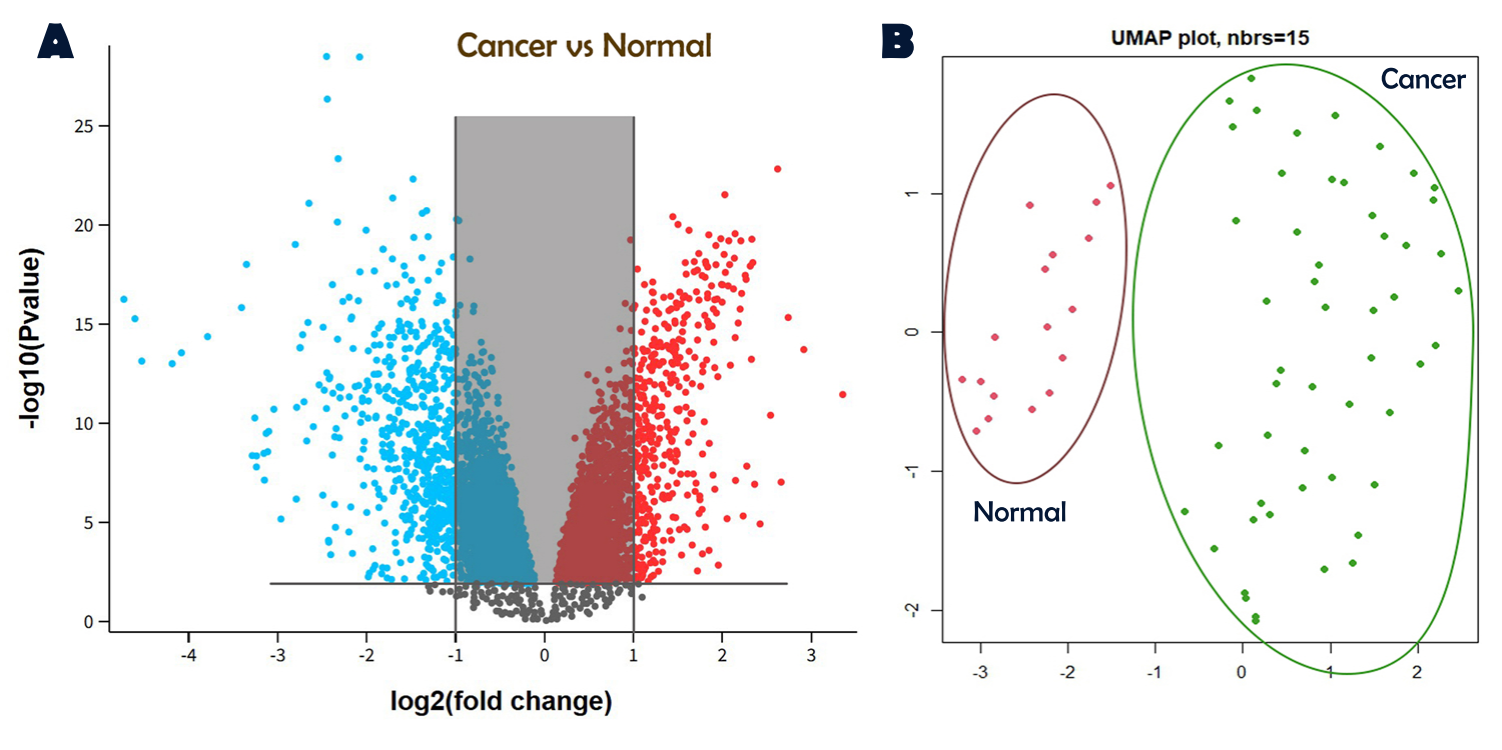
Identification of DEGs between Normal and Tumor Samples. (A) Volcano plot illustrating the DEGs identified in the GSE52903 dataset. (B) UMAP (Uniform Manifold Approximation and Projection) visualization of the sample distribution, showcasing the clustering and separation of normal and tumor samples based on gene expression profiles

Figure 1 illustrates the identification of DEGs through a volcano plot (Figure 1A) and the dimensional distribution of samples using UMAP (Figure 1B). In GSE52903 as the main dataset, 917 DEGs containing 347 upregulated and 570 downregulated genes. In the validation datasets, 813 DEGs were screened for GSE7410 and 887 DEGs for GSE 29570.

###  GO and KEGG Pathway Analysis

**Figure 2:**
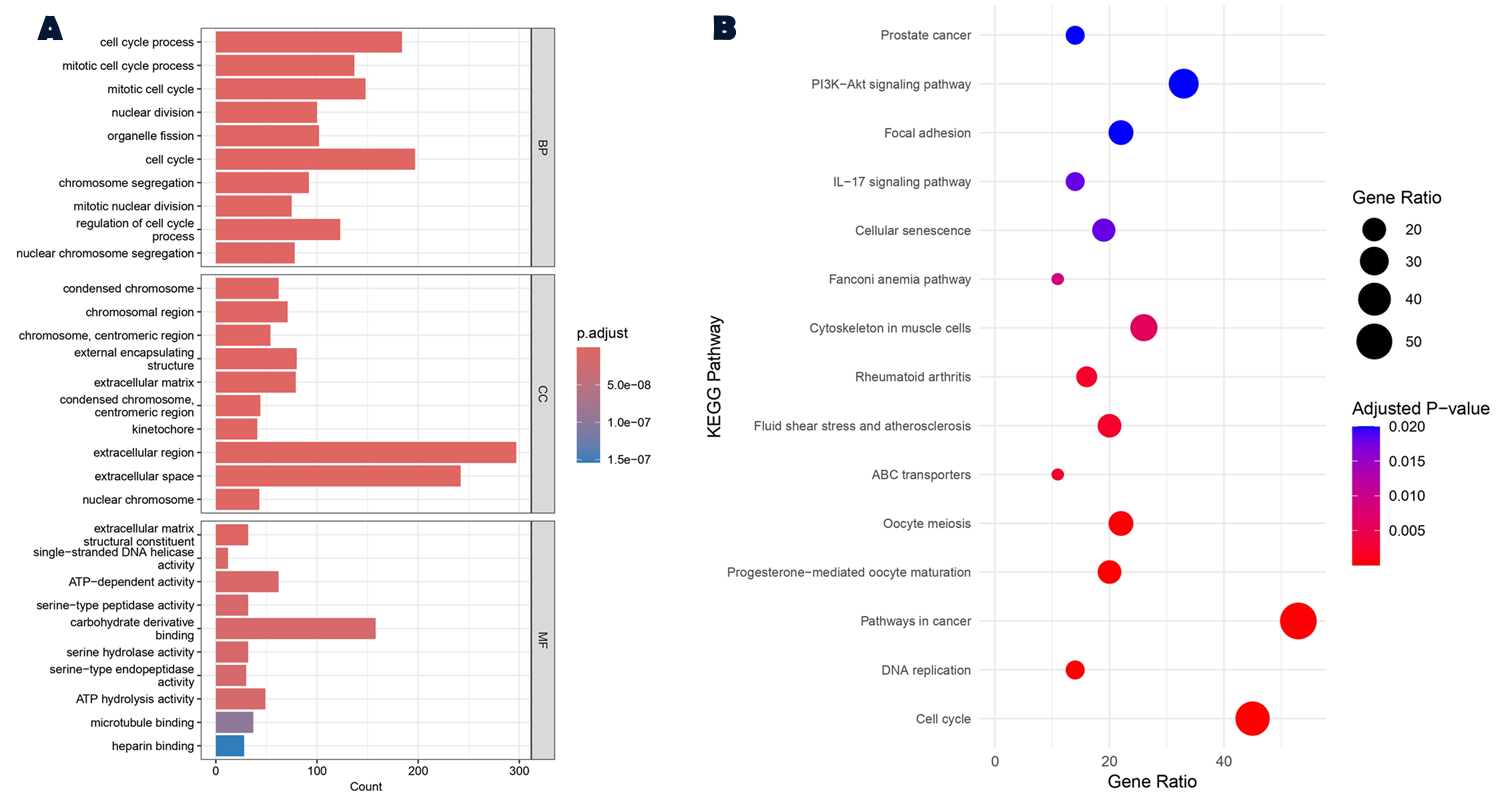
Gene Ontology and KEGG Pathway Enrichment Analyses of DEGs Using ClusterProfiler. (A) Results of GO enrichment analysis, categorized into biological processes, cellular components, and molecular functions. (B) KEGG pathway enrichment analysis highlighting the significant pathways associated with the identified DEGs

 KEGG pathway analysis revealed that “cell cycle”, “pathways in cancer”, “oocyte meiosis” and “PI3K-Akt signaling pathway” are among the most important pathways related to the screened DEGs. Additionally, GO analysis, which classifies genes into three categories (molecular function (MF), biological process (BP), and cellular component (CC)) showed that DEGs were more strongly related to BP of “cell cycle process” (GO:0022402), CC of “condensed chromosome” (GO:0000793) and MF of “extracellular matrix structural constituent” (GO:0005201) (Figure 2).

###  Exploring Diagnostic Biomarkers: Feature Selection using ML

 Feature selection was performed using a GA to identify the most significant DEGs associated with cervical cancer. GA was executed 100 times, with each run comprising 100 generations. During each generation, potential gene subsets were evaluated based on their classification performance using a SVM as the fitness function. From these runs, 8000 combinations of the best-performing gene subsets were extracted. Subsequently, to achieve 100% classification accuracy, the top eight genes with the highest selection frequency each appearing more than 4000 times across the 8000 selected combinations were chosen as the most significant features. The final SVM model (with linear kernel) was evaluated 50 times using these selected genes, with 5-fold cross-validation. The accuracy results are summarized in Table 1.

**Table 1:** Comparison of Classification Performance on the GSE52903 Dataset Using Different Approaches

**Method**	**Accuracy %**	**Precision %**	**Recall %**	**F1 score %**	**ROC AUC %**	**Number of selected features**
SVM-GA Best practice	100	100	100	100	100	8
SVM-GA^*^ (Mean ± SD)	98.90 ± 0.60	99.22 ± 0.93	99.36 ± 0.88	99.28 ± 0.38	99.0 ± 0.12	8
SVM	98.61	98.18	100	99.08	99.09	917
ANN	97.22	98.18	98.18	98.18	96.15	917

SVM, support vector machine; GA, genetic algorithm; ANN, artificial neural network; SD, standard deviation; ROC AUC, area under the ROC curve. *Result is presented as mean ± SD in 50 repeats.

 For validation, two independent datasets, GSE29570 and GSE7410, were used. The eight selected genes “CXCL9, CTGF, ZNF704, ZEB2, SASH1, PTN, KPNA2, SLC5A1” were evaluated in these datasets, and the SVM model was applied to classify tumor and normal samples. The results of this validation process, including model accuracy and performance metrics, are presented in Table 2.

**Table 2:** Performance Evaluation of the SVM Model Using the Eight Selected Genes on the Validation Datasets (GSE29570 and GSE7410)

**Data set**	**Accuracy %**	**Precision %**	**Recall %**	**F1 score %**
GSE29570	98.80 ± 1.0	99.50 ± 1.0	98.8 ± 1.0	99.1 ± 1.0
GSE7410	100 ± 0	100 ± 0	100 ± 0	100 ± 0

GSE, Gene Expression Omnibus Series. Results are presented as Mean ± SD in 100 repeats.

###  Identification of Therapeutic Targets Based on PPI Network

 In this study, 508 genes that were selected more than 3000 times by the applied model were subjected to PPI network analysis. Based on the defined criteria, 42 genes were identified as key nodes in the network, with the corresponding PPI network presented in (Supplementary file 1, Figure S1). We consider these genes as potential therapeutic targets for cervical cancer, given their central roles and high connectivity within the PPI network. These genes, selected based on their prominent interactions, represent promising candidates for further investigation in the development of targeted therapies for cervical cancer.

 Among these selected genes, CDK1, BRCA1, CCNB1, BIRC5, CHEK1, RAD51, AURKB, AURKA, and BUB1 demonstrated the highest degree of connectivity, highlighting their central roles in the network.

###  Survival Analysis of Key Genes Selected from the Network for Identification of Biomarkers with Prognostic Value 

**Figure 3:**
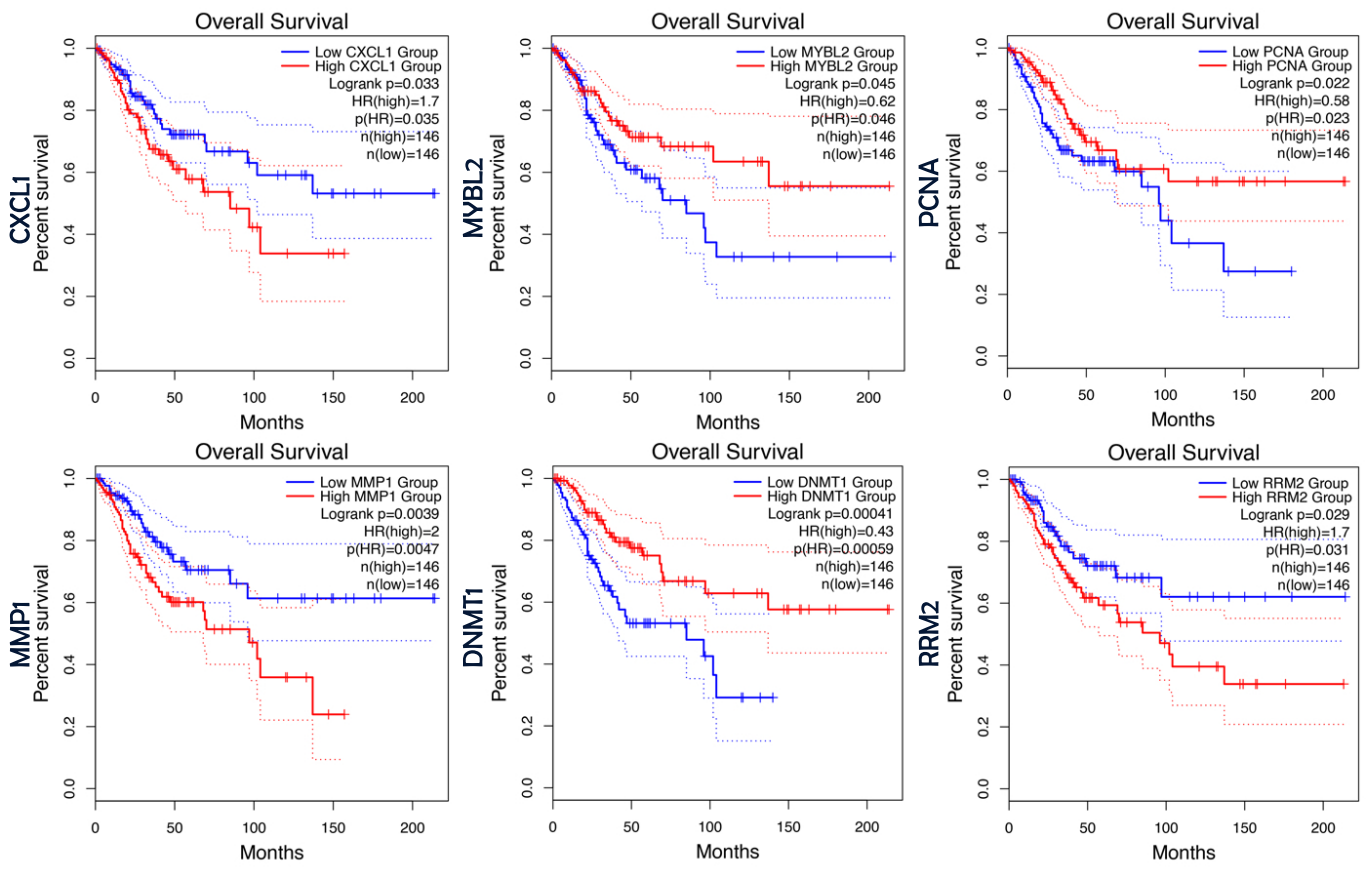
Survival Analysis of Therapeutic Targets. This figure presents Kaplan-Meier plots illustrating the survival analysis of the therapeutic target genes. The figure highlights the plots for genes with significant prognostic value including CXCL1, MYBL2, PCNA, MMP1, DNMT1 and RRM2 as determined overall survival analysis in GEPIA

 Survival analysis was conducted for the 42 genes identified in the previous step using the GEPIA platform. Among these genes, six (CXCL1, DNMT1, MMP1, MYBL2, PCNA, and RRM2) were identified as having statistically significant prognostic value based on this criterion (Figure 3). These genes were subsequently selected for further investigation as potential prognostic biomarkers.

###  Expression and Immunohistochemistry Validation of Prognostic Biomarkers In Silico

 Using the GEPIA platform, we validated the expression levels of the selected genes between normal and cervical cancer samples. The analysis revealed that among the six final prognostic biomarkers, CXCL1, MMP1, MYBL2, PCNA, and RRM2 exhibited significant overexpression in cervical cancer tissues compared to normal tissues (Supplementary File 1, Figure S2 A). Among these, the protein expression levels of four hub genes (excluding CXCL1 and MMP1, for which no IHC data was available) were notably higher in normal cervix tissues compared to cervical cancer tissues, corroborating the findings from the gene expression analysis (Supplementary file 1, Figure S2 B).

###  Transcription Factors Modulating Prognostic Biomarkers

 The TF–DEGs network was constructed using the NetworkAnalyst tool and ENCODE database. According to this database, a total of 51 TFs were found to be related to the genes. Among these TFs, E2F1 and TP63 were related to five of these six gene regulating them and can be considered as the most important TFs. In addition, MYC, BACH1, FOXA1, KLF4, EP300 and POU5F1 were other important TFs in the constructed network. The network is shown in (Supplementary file 1, Figure S3).

## Discussion

 This research introduced a hybrid machine learning model designed to accurately predict cervical cancer, using gene expression data from human samples. The findings demonstrated that the proposed model effectively distinguished between cervical cancer cases and healthy controls. To assess its performance, the predicted outcomes (binary classification: cervical cancer vs. control) from the model during the validation phase (test set) were compared to the actual known diagnoses (true binary response: cervical cancer vs. control). A high AUC and accuracy would indicate an optimal prediction model. Additionally, a traditional SVM (without GA) and ANN were trained and compared with the hybrid model. The results indicated that the proposed hybrid model outperformed the traditional SVM and ANN, with GA significantly enhancing the SVM classifier’s performance, achieving an impressive accuracy rate of 99%. Moreover, the application of a GA for feature selection proved highly effective in identifying the most relevant genes associated with cervical cancer. This is evident from the validation results, where the selected eight genes enabled the SVM model to achieve high accuracy in predicting outcomes on independent datasets (GSE29570 and GSE7410). The GA successfully reduced the dimensionality of the data while retaining the most informative features, allowing the classification model to perform at its best. Furthermore, the SVM classifier demonstrated excellent performance, particularly when used in conjunction with the GA-selected features. By reducing the number of features, the model not only maintained its predictive accuracy but also exhibited an improvement compared to the scenario where no feature selection was applied. This highlights the impact of dimensionality reduction in mitigating overfitting and enhancing the model’s ability to generalize across datasets. These findings emphasize the robustness and potential of combining GA-based feature selection with SVM for biomarker identification and classification tasks in biomedical studies.

 Similar findings have been reported in other studies, where the combination of GA with SVM has proven effective in feature selection and improving classification accuracy in cancer research. For instance, a study by Huerta et al. demonstrated the effectiveness of the GA-SVM approach in gene selection and microarray data classification.^20^ Similarly, Tapak et al applied GA-SVM in identifying gene expression signatures for disease classification, showing enhanced performance compared to traditional methods.^30^

 These findings emphasize the robustness and potential of combining GA-based feature selection with SVM for biomarker identification and classification tasks in biomedical studies.

 The eight genes (CXCL9, CTGF, ZNF704, ZEB2, SASH1, PTN, KPNA2, and SLC5A1) were selected through our hybrid model approach as potential diagnostic biomarkers for cervical cancer. These genes, which were selected more than 4000 time in our feature selection method, have been implicated in various molecular processes associated with the development and progression of cervical cancer, including immune response regulation, tumor progression, metastasis, and cellular signaling. Each of these genes plays a crucial role in the disease, and their potential as diagnostic markers lies in the mechanisms through which they contribute to the pathogenesis of cervical cancer. Further details on the genes are provided in (Supplementary file 2).

 In the next section of our study, we focused on identifying biomarkers with prognostic value in cervical cancer. To achieve this, we systematically evaluated the 42 therapeutic targets identified in the previous step to determine which ones also possess prognostic significance. By incorporating this additional filtering step, we strengthened our analysis by narrowing down the candidate genes to those that are not only therapeutically relevant but also hold prognostic value. Among the 42 therapeutic targets examined, six genes (CXCL1, DNMT1, MMP1, MYBL2, PCNA, and RRM2) demonstrated statistically a significant prognostic value. Notably, DNMT1 and MMP1 emerged as the most significant prognostic markers, with log-rank *P* values of 0.00041 and 0.0039, respectively.

 DNMT1 and MMP1 play crucial roles in cervical cancer progression and prognosis. Guo et al found that DNMT1 expression is significantly elevated in cervical cancer tissues compared to normal tissues, correlating with pathological stage, lymph node metastasis, and high-risk HPV infection.^31,32^ Higher DNMT1 expression was associated with lower 3-year survival rates and showed a strong correlation with galectin-1 levels, suggesting its potential as a prognostic marker.^32^ Similarly, MMP1 has been linked to lymph node metastasis and poor survival outcomes. A meta-analysis of 18 studies confirmed that MMP overexpression, including MMP1, is associated with reduced overall and recurrence-free survival in cervical cancer patients.^33-35^ Persistent MMP1 overexpression in metastatic samples highlights its role in tumor progression and its potential as a biomarker for disease severity and metastatic risk. Further studies are needed to validate its clinical utility.

 The next phase of our study focused on identifying key transcription factors that regulate the final six genes selected in the previous step, which serve as both prognostic markers and therapeutic targets. This step was crucial for uncovering the downstream regulatory mechanisms governing the expression of these genes. E2F1 and TP63 were identified as the most significant transcription factors modulating these genes, playing a crucial role in regulating their expression and influencing the molecular pathways associated with cervical cancer progression.

 E2F1 and TP63 are important players in the progression of cervical cancer. E2F1, the one often upregulated in high-risk HPV infections, promotes tumor growth and migration by classical target genes, including TOP2A, BIRC5, MDM2, and MELK.^36-38^ Because of its central role in cancer development, the targeting of E2F1 and downstream pathways represents potential new therapeutic approaches. Likewise, TP63 is a member of the p53 family with two main isoforms: TAp63 and ΔNp63, with opposing impacts in a tumor. Increased ratios of ΔNp63 to TAp63 expression are associated with the progression of cervical intraepithelial neoplasia into invasive cancers.^39,40^ In HPV-positive patients, the degree of TP63 promoter methylation further correlates with the severity of lesions, supporting its potential as a diagnostic and prognostic marker.^41^ Such knowledge further elucidates the importance of E2F1 and TP63 as possible molecular targets for improving the diagnosis and treatment of cervical cancer.

 While our study presents promising results in identifying novel key genes for cervical cancer diagnosis and prognosis through a hybrid machine learning approach, we acknowledge certain limitations that may affect the robustness and generalizability of our findings. One such limitation is the relatively small sample size for some groups, particularly the normal tissue samples. To mitigate this, we validated our findings using multiple external datasets, including the TCGA dataset, to ensure the robustness and generalizability of the identified biomarkers.

 Additionally, although we performed *in-silico* validation using gene expression and PPI data, our study lacks experimental validation, which is crucial for confirming the functional roles of the identified biomarkers in cervical cancer progression. Further research involving *in vitro* and *in vivo* validation of these genes is needed to fully establish their potential as therapeutic targets.

 Another consideration is the use of a single dataset as the main dataset in our study. While this approach helped to prevent batch effects that could arise from merging multiple datasets, it also allowed for a more focused and controlled analysis.^42^ To ensure the generalizability of our findings, we validated the results using multiple external datasets. This strategy mitigated potential biases and reinforced the robustness and reliability of our results, demonstrating the effectiveness of hybrid machine learning algorithms in providing consistent and accurate insights across different data sources.

 Furthermore, cervical cancer is a highly heterogeneous disease, with multiple molecular subtypes and diverse pathways contributing to its progression. While our study focused on key genes and pathways, it may not fully capture the complexity of the disease. Incorporating multi-omics data, such as genomics, proteomics, and epigenomics, could offer a more comprehensive understanding of cervical cancer biology and improve the identification of more accurate biomarkers for diagnosis and prognosis.

## Conclusion

 In this study, we applied a hybrid machine learning approach combining GA and SVM to identify key genes linked to cervical cancer. Eight significant genes (CXCL9, CTGF, ZNF704, ZEB2, SASH1, PTN, KPNA2, and SLC5A1) were identified as potential diagnostic biomarkers, involved in immune regulation, tumor progression, and metastasis. The hybrid SVM-GA model achieved 99% accuracy in classifying cancerous tissues, demonstrating its potential for early detection. PPI analysis revealed 42 therapeutic targets and survival analysis revealed prognostic genes, identifying CXCL1, DNMT1, MMP1, MYBL2, PCNA, and RRM2 as key therapeutic targets with a significant prognostic value. Additionally, transcription factor analysis highlighted E2F1 and TP63 as key regulators. The identified genes and pathways offer valuable targets for personalized treatment approaches, with the potential to improve patient outcomes. Future research should focus on validating these biomarkers in larger, diverse patient populations to fully explore their clinical utility.

## Supplementary files

Supplementary file 1. Supplementary Network Analyses (Figures S1, S2, and S3).

Supplementary file 2. Literature Review on Selected Genes.
