# Supplementary material for: In Silico Transcriptomic Analysis for Identification of Potential Diagnostic and Prognostic Biomarkers and Therapeutic Targets in Cervical Cancer using a Hybrid Genetic Algorithm–Support Vector Machine Approach
Source: Arch Iran Med. 2025 Dec 1;28(12):677–86. doi: 10.34172/aim.34814 (PMC13000335; doi:10.34172/aim.34814)
Supplement: Supplementary file 1 — Supplementary Network Analyses (Figures S1, S2, and S3). [file aim-28-677-s001.pdf]

Arch Iran Med. December 2025;28(12):677-S1

10.34172/aim.34814

http://journalaim.com

Supplementary Network Analyses

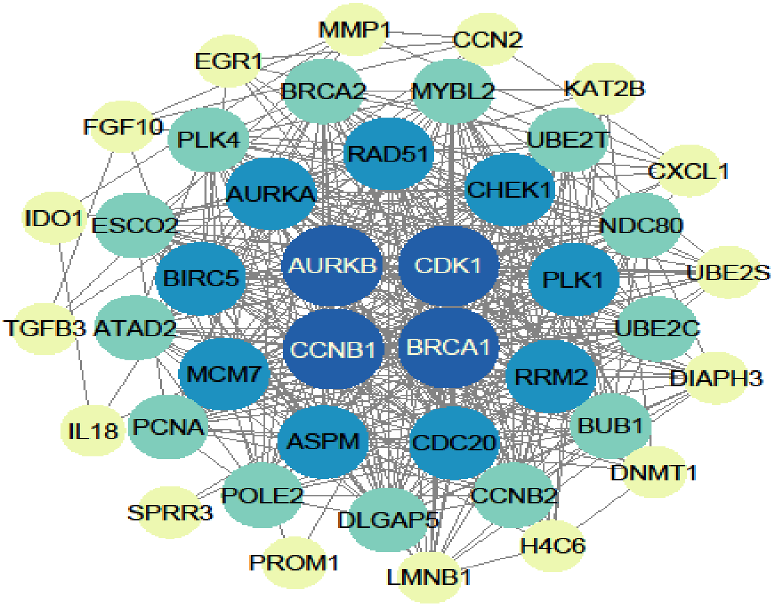

Figure S1. PPI network of therapeutic targets. This figure illustrates the PPI network constructed from 42 genes with the highest degrees, identified based on criteria such as betweenness centrality and node degree. These genes are proposed as potential therapeutic targets for cervical cancer.

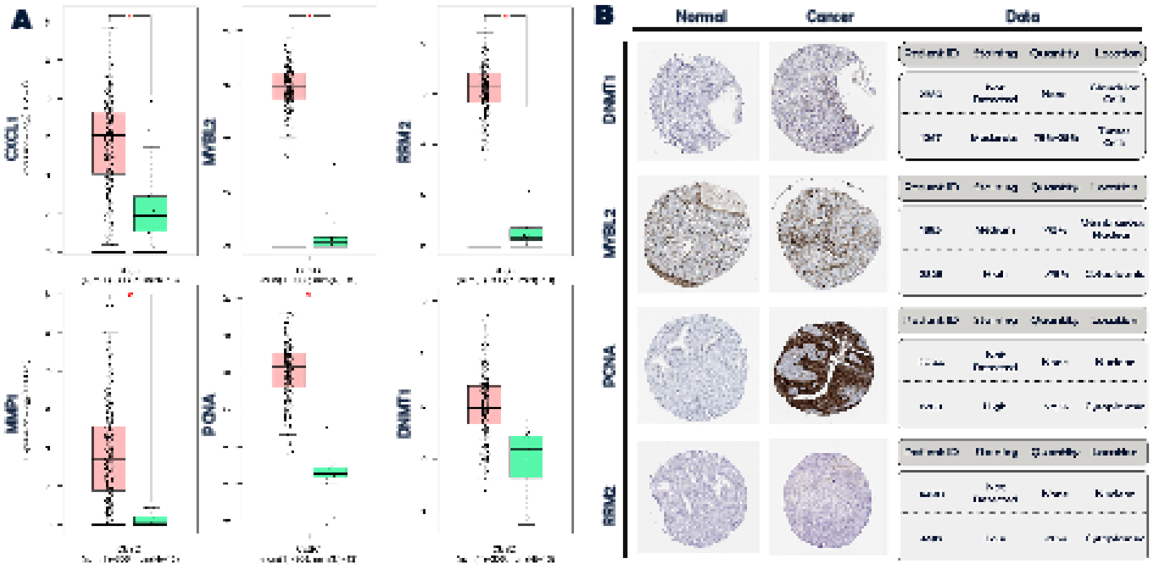

Figure S2. Validation of six prognostic genes using the TCGA-CESC database and immunohistochemistry data. (A) Expression levels of CXCL1, MYBL2, PCNA, MMP1, DNMT1, and RRM2 in cervical cancer samples compared to normal samples. (B) Protein expression validation for MYBL2, PCNA, DNMT1, and RRM2 in cervical cancer tissues versus normal tissues, using immunohistochemistry images from the Human Protein Atlas database.

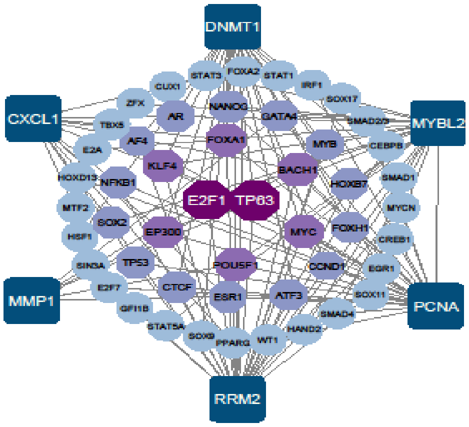

Figure S3. TF–DEGs Interaction Network. This figure depicts the transcription factor (TF)–DEG interaction network, highlighting interactions between 65 TFs and the six final prognostic genes. Among these, E2F1 and TP63 interact with five genes, making them the most significant TFs within the network. TFs are color-coded to represent their level of interaction: purple for TFs interacting with five genes, lavender for four genes, indigo for three genes, and light blue for those connected to one or two genes. The genes are displayed as blue squares. This network was constructed using Cytoscape software.
