# Supplementary material for: In Silico Transcriptomic Analysis for Identification of Potential Diagnostic and Prognostic Biomarkers and Therapeutic Targets in Cervical Cancer using a Hybrid Genetic Algorithm–Support Vector Machine Approach
Source: Arch Iran Med. 2025 Dec 1;28(12):677–86. doi: 10.34172/aim.34814 (PMC13000335; doi:10.34172/aim.34814)
Supplement: Supplementary file 2 — Literature Review on Selected Genes. [file aim-28-677-s002.pdf]

Arch Iran Med. December 2025;28(12):677-S2
10.34172/aim.34814
http://journalaim.com

Literature Review on Selected Genes

CXCL9, a member of the CXC chemokine family, plays a key role in immune cell chemotaxis, recruiting various immune and non-immune cells, including T lymphocytes, NK cells, dendritic cells, macrophages, and fibroblasts.[1] Its complex role in tumors has made it an important prognostic marker, with its expression linked to tumor progression and patient outcomes.[2] Specifically in cervical cancer, Zhi et al. found significantly elevated serum CXCL9 levels in women with invasive stages (II and III) compared to those with non-invasive stages, suggesting its involvement in cancer development.[3] Furthermore, CXCL9 has been shown to correlate with immune cells like CD8+ T cells, NK cells, and Th1 cells in the tumor microenvironment, highlighting its potential in mediating anti-tumor immune responses.[4]

Connective tissue growth factor (CTGF), a member of the CCN family of matrix-associated proteins, plays a significant role in various biological processes, including angiogenesis and tumor progression.[5] CTGF is involved in regulating key cellular functions such as proliferation, drug resistance, angiogenesis, adhesion, and migration, all of which contribute to metastasis. The expression pattern of CTGF in tumors has been linked to tumor development and response to therapy, providing insights into its role across different types of cancer.[6] Hofsjö et al. found elevated CTGF mRNA expression in the vaginal wall of cancer survivors compared to controls, highlighting its involvement in tissue remodeling.[7] This finding aligns with our earlier observation of radiotherapy-induced fibrosis and elastosis in the connective tissue of the vaginal wall in cervical cancer survivors, further emphasizing the significance of CTGF in cancer-related tissue alterations.[6,7]

Zinc finger protein 704 (ZNF704), a member of the zinc-finger protein family located on chromosome 8q21, has been implicated in the promotion of carcinogenesis in various cancers.[8] Previous studies have shown that ZNF704 upregulation enhances tumorigenesis in breast cancer. In the context of cervical cancer, ZNF704 is overexpressed and plays a critical role in regulating the cell cycle and promoting tumor cell survival.[9]

ZEB2 (zinc finger E-box binding homeobox 2) is a transcription factor that plays a pivotal role in epithelial-to-mesenchymal transition (EMT), a crucial process in cancer metastasic.[10] In cervical cancer, ZEB2 promotes the invasive and migratory behavior of tumor cells, contributing to disease progression. Recent studies have further elucidated that ZEB2 acts as a downstream target of miR-505, and its metastatic effects are mediated through the lncRNA-CTS/miR-505/ZEB2 signaling axis in cervical cancer cells.[11,12]

The SASH1 gene, a member of the SLY-family of signal adapter proteins, encodes a protein involved in intracellular signaling and has been identified as a potential tumor suppressor in multiple cancers.[13] Studies indicate that SASH1 is significantly downregulated in cervical, breast,

and gastric cancers, with its overexpression inhibiting cancer cell proliferation and invasion. In cervical cancer, SASH1 suppresses the FAK signaling pathway[13], while in breast cancer, it inhibits the PI3K-Akt-mTOR pathway, correlating with improved overall and recurrence-free survival. In gastric cancer, low SASH1 expression is associated with advanced TNM stage and poor prognosis, highlighting its potential as an independent prognostic biomarker.[14] Collectively, these findings suggest that SASH1 plays a crucial tumor-suppressive role by modulating key oncogenic pathways, making it a promising therapeutic target and biomarker in cancer research.

Pleiotrophin (PTN) is a heparin-binding cytokine implicated in various cellular functions, including glial differentiation, neurite outgrowth, and angiogenesis. Studies indicate that PTN is significantly overexpressed in cervical cancer tissues compared to normal tissues though its expression is not correlated with tumor stage or size.[15] A meta-analysis by Zhou et al. confirms that high PTN expression is associated with advanced TNM stage and poor overall survival but not with tumor size, lymph node metastasis, or histological grade.[16] In ovarian cancer, PTN has been identified as a molecular vulnerability, with siRNA-mediated knockdown inducing apoptosis in epithelial ovarian cancer (EOC) cells.[17]

Karyopherin α2 (KPNA2), a member of the nuclear transporter family, is overexpressed in various cancers, including cervical and ovarian cancers, and is associated with poor prognosis.[18] In cervical cancer, high KPNA2 expression correlates with advanced tumor stage, larger tumor size, and poorer overall and recurrence-free survival. KPNA2 downregulation serves as an independent prognostic marker, suggesting its role as an oncogene.[19] In ovarian cancer, KPNA2 overexpression promotes cell proliferation and tumorigenicity by driving the G1/S cell cycle transition, enhancing c-Myc and Akt activity, and regulating key cyclin-dependent kinases. Knockdown of KPNA2 suppresses these processes, indicating its importance in tumor progression.[20] These findings position KPNA2 as a potential prognostic biomarker and therapeutic target for cervical and ovarian cancers.

SLC5A1 (solute carrier family 5 member 1) encodes a sodium-dependent glucose transporter (SGLT) involved in glucose and galactose uptake from the intestine. Aberrant expression of SLC5A1 has been reported as a negative prognostic factor in various cancers, including pancreatic cancer, where it is overexpressed in both tissues and cell lines. Mechanistically, SLC5A1 inhibition leads to AMPK-dependent mTOR suppression, with AMPK inhibition reversing the effects of SLC5A1 blockade. Additionally, SLC5A1 interacts with EGFR, and its knockdown, along with EGFR inhibition, results in decreased cell survival and glucose uptake. These findings suggest that the SLC5A1/EGFR pathway could be a potential therapeutic target for pancreatic cancer.[21] Although no studies have directly linked SLC5A1 to cervical cancer, our research suggests that this gene may be associated with the diagnosis of cervical cancer, highlighting its potential relevance in the context of this disease.

In the next phase of our research, we identified 42 key genes as potential therapeutic targets for cervical cancer by analyzing a PPI network of 508 genes selected over 3,000 times in our hybrid model. Among them, CDK1, BRCA1, CCNB1, and AURKB showed the highest connectivity, highlighting their central roles in the network. CDK1 inhibitors have been explored in clinical trials as potential cancer therapies, with notable candidates including BEY1107, flavopiridol, roniciclib, P276-00, dinaciclib, and AT7519. These inhibitors have shown varying degrees of

efficacy in treating cancers such as leukemia, pancreatic cancer, multiple myeloma, and lung cancer.[22] while no studies have specifically evaluated CDK1 inhibitors in cervical cancer, their mechanisms suggest potential utility in this malignancy, warranting further investigation.

BRCA1, CCNB1, and AURKs play significant roles in cancer progression and therapy. BRCA1, a tumor suppressor gene, maintains genomic stability by regulating DNA damage response, transcription, and cell cycle checkpoints. Its dysfunction contributes to various cancers, including breast, ovarian, and pancreatic malignancies, making it a key therapeutic target. PARP inhibitors and emerging approaches such as DDR inhibitors and immunotherapy are being explored to improve treatment strategies.[23,24] CCNB1, a cyclin family member, is overexpressed in multiple cancers and serves as a prognostic biomarker in ER+ breast cancer and cervical cancer. Its overexpression is linked to poor prognosis, increased proliferation, and therapy resistance. Targeting the CCNB1/CDK1 pathway through inhibitors and combination therapies offers potential therapeutic benefits.[25-27]

Additionally, HPV oncogene E7 sensitizes cells to aurora kinase (AURK) inhibition, particularly AURKB, which is crucial for mitotic progression. Inhibition of AURKB with MLN8237 (Alisertib) disrupts mitosis in HPV-positive cells, leading to apoptosis and tumor reduction, making AURK inhibitors a promising treatment strategy for HPV-driven cancers.[28,29]

References

1. Ding Q, Lu P, Xia Y, Ding S, Fan Y, Li X, et al. CXCL9: evidence and contradictions for its role in tumor progression. Cancer Med. 2016;5(11):3246-59. doi: 10.1002/cam4.934.
2. Zhang Y, Sun B, Hu M, Lou Y, Lu J, Zhang X, et al. CXCL9 as a prognostic inflammatory marker in early-stage lung adenocarcinoma patients. Front Oncol. 2020;10:1049. doi: 10.3389/fonc.2020.01049.
3. Zhi W, Ferris D, Sharma A, Purohit S, Santos C, He M, et al. Twelve serum proteins progressively increase with disease stage in squamous cell cervical cancer patients. Int J Gynecol Cancer. 2014;24(6). doi: 10.1097/IGC.0000000000000153.
4. Kong W, Zhao G, Chen H, Wang W, Shang X, Sun Q, et al. Analysis of therapeutic targets and prognostic biomarkers of CXC chemokines in cervical cancer microenvironment. Cancer Cell Int. 2021;21(1):399. doi: 10.1186/s12935-021-02101-9.
5. Wells JE, Howlett M, Cole CH, Kees UR. Deregulated expression of connective tissue growth factor (CTGF/CCN2) is linked to poor outcome in human cancer. Int J Cancer. 2015;137(3):504-11. doi: 10.1002/ijc.28972.
6. Chu C-Y, Chang C-C, Prakash E, Kuo M-L. Connective tissue growth factor (CTGF) and cancer progression. Journal of Biomedical Science. 2008;15(6):675-85. doi: 10.1007/s11373-008-9264-9.
7. Hofsjö A, Bohm-Starke N, Bergmark K, Masironi B, Sahlin L. Sex steroid hormone receptor expression in the vaginal wall in cervical cancer survivors after radiotherapy. Acta Oncol. 2019;58(8):1107-15. doi: 10.1080/0284186X.2019.1598574.
8. Jia Z, Wang F,Gongzhuo L, Jiang P, Leng Y, Ke L, et al. Zinc finger protein 468 up-regulation of TFAM contributes to the malignant growth and cisplatin resistance of breast cancer cells. Cell Div. 2024;19(1):8. doi: 10.1186/s13008-024-00113-1.
9. Luo J, Li H, Xiu J, Zeng J, Feng Z, Zhao H, et al. Elevated ZNF704 expression is associated with poor prognosis of uveal melanoma and promotes cancer cell growth by regulating AKT/mTOR signaling. Biomark Res. 2023;11(1):38. doi: 10.1186/s40364-023-00471-y.

10. Fang S, Zeng X, Zhu W, Tang R, Chao Y, Guo L. Zinc finger E-box-binding homeobox 2 (ZEB2) regulated by miR-200b contributes to multi-drug resistance of small cell lung cancer. Exp Mol Pathol. 2014;96(3):438-44. doi: 10.1016/j.yexmp.2014.04.008.
11. Wang W, Xu A, Zhao M, Sun J, Gao L. Circ_0001247 functions as a miR-1270 sponge to accelerate cervical cancer progression by up-regulating ZEB2 expression level. Biotechnol Lett. 2021;43(3):745-55. doi: 10.1007/s10529-020-03059-w.
12. Feng S, Liu W, Bai X, Pan W, Jia Z, Zhang S, et al. LncRNA-CTS promotes metastasis and epithelial-to-mesenchymal transition through regulating miR-505/ZEB2 axis in cervical cancer. Cancer Lett. 2019;465:105-17. doi: 10.1016/j.canlet.2019.09.002.
13. Chen H, Wang D, Liu Y. SASH1 inhibits cervical cancer cell proliferation and invasion by suppressing the FAK pathway. Mol Med Rep. 2016;13(4):3613-8. doi: 10.3892/mmr.2016.4946.
14. Zhou N, Liu C, Wang X, Mao Q, Jin Q, Li P. Downregulated SASH1 expression indicates poor clinical prognosis in gastric cancer. Hum Pathol. 2018;74:83-91. doi: 10.1016/j.humpath.2018.01.008.
15. Deuel TF, Zhang N, Yeh H-J, Silos-Santiago I, Wang Z-Y. Pleiotrophin: A Cytokine with Diverse Functions and a Novel Signaling Pathway. Arch Biochem Biophys. 2002;397(2):162-71. doi: 10.1006/abbi.2001.2705.
16. Zhou J, Yang Y, Zhang Y, Liu H, Dou Q. A meta-analysis on the role of pleiotrophin (PTN) as a prognostic factor in cancer. PLOS ONE. 2018;13(11):e0207473. doi: 10.1371/journal.pone.0207473.
17. Sethi G, Kwon Y, Burkhalter RJ, Pathak HB, Madan R, McHugh S, et al. PTN signaling: Components and mechanistic insights in human ovarian cancer. Mol carcinog. 2015;54(12):1772-85. doi: 10.1002/mc.22249.
18. Han Y, Wang X. The emerging roles of KPNA2 in cancer. Life Sci. 2020;241:117140. doi: 10.1016/j.lfs.2019.117140.
19. Kang N, Chen P. Overexpression of KPNA2 is associated with unfavorable prognosis in cervical carcinoma. Int J Clin Exp Med. 2016;9(3):6316-22.
20. Huang L, Wang HY, Li JD, Wang JH, Zhou Y, Luo RZ, et al. KPNA2 promotes cell proliferation and tumorigenicity in epithelial ovarian carcinoma through upregulation of c-Myc and downregulation of FOXO3a. Cell Death Dis. 2013;4(8):e745. doi: 10.1038/cddis.2013.256.
21. Gao H-F, Chen L-Y, Cheng C-S, Chen H, Meng Z-Q, Chen Z. SLC5A1 promotes growth and proliferation of pancreatic carcinoma via glucose-dependent AMPK/mTOR signaling. Cancer Manag Res. 2019;11:3171-85. doi: 10.2147/CMAR.S195424.
22. Wang Q, Bode AM, Zhang T. Targeting CDK1 in cancer: mechanisms and implications. NPJ Precis Oncol. 2023;7(1):58. doi: 10.1038/s41698-023-00407-7.
23. Raimundo L, Calheiros J, Saraiva L. Exploiting DNA Damage Repair in Precision Cancer Therapy: BRCA1 as a Prime Therapeutic Target. Cancers. 2021;13(14):3438. doi: 10.3390/cancers13143438.
24. Romagnolo AP, Romagnolo DF, Selmin OI. BRCA1 as target for breast cancer prevention and therapy. Anticancer Agents Med Chem 2015;15(1):4-14. doi: 10.2174/1871520614666141020153543.
25. Zhang H, Zhang X, Li X, Meng WB, Bai ZT, Rui SZ, et al. Effect of CCNB1 silencing on cell cycle, senescence, and apoptosis through the p53 signaling pathway in pancreatic cancer. J Cell Physiol. 2018;234(1):619-31. doi: 10.1002/jcp.26816.
26. Ding K, Li W, Zou Z, Zou X, Wang C. CCNB1 is a prognostic biomarker for ER+ breast cancer. Med Hypotheses. 2014;83(3):359-64. doi: 10.1016/j.mehy.2014.06.013.
27. Fang L, Du WW, Awan FM, Dong J, Yang BB. The circular RNA circ-Ccnb1 dissociates Ccnb1/Cdk1 complex suppressing cell invasion and tumorigenesis. Cancer Lett. 2019;459:216-26. doi: 10.1016/j.canlet.2019.05.036.
28. Yumol J, Gabrielli B, Tayyar Y, McMillan NA, Idris A. Smart drug combinations for cervical cancer: dual targeting of Bcl-2 family of proteins and aurora kinases. Ame J Cancer Res. 2020;10(10):3406-14.

29. Gabrielli B, Bokhari F, Ranall MV, Oo ZY, Stevenson AJ, Wang W, et al. Aurora A Is Critical for Survival in HPV-Transformed Cervical Cancer. Mol Cancer Ther. 2015;14(12):2753-61. doi: 10.1158/1535-7163.MCT-15-0506.
